# Impaired Fertility and Sexual Function in Women With Hirschsprung Disease: Results From an International Multi‐Centre Cross‐Sectional Study

**DOI:** 10.1111/1471-0528.18294

**Published:** 2025-07-10

**Authors:** Joseph R. Davidson, Annika Mutanen, Anna Löf Granström, Anders Telle Hoel, Stavros P. Loukogeorgakis, Paolo De Coppi, Kristin Bjørnland, Tomas Wester, Simon Eaton, Mikko P. Pakarinen, Joe Curry

**Affiliations:** ^1^ Great Ormond Street Hospital for Children London UK; ^2^ UCL Great Ormond Street Institute of Child Health London UK; ^3^ New Children's Hospital Helsinki Finland; ^4^ University of Helsinki Helsinki Finland; ^5^ Karolinska University Hospital Stockholm Sweden; ^6^ Karolinska Institutet Stockholm Sweden; ^7^ Oslo University Hospital Oslo Norway; ^8^ University of Oslo Oslo Norway

**Keywords:** bowel incontinence, congenital colorectal disease, fertility outcomes, FSFI, Hirschsprung disease, HSCR, sexual function, subfertility

## Abstract

**Objective:**

Hirschsprung is a congenital disorder affecting the gastrointestinal tract. However, pelvic colorectal surgery in infancy has been hypothesised to impact gynaecological outcomes in later life. Describe sexual function and fertility outcomes in women with Hirschsprung disease compared to population controls. Assess factors associated with poor outcomes (sexual dysfunction and subfertility).

**Design:**

International multicentre cross‐sectional cohort study with comparison to controls from the general population.

**Setting:**

Status post‐discharge from paediatric services.

**Population:**

Female patients aged > 20 years.

**Methods:**

Validated questionnaire‐based survey with linkage to patient medical records. Comparison with controls using univariate analyses.

**Main Outcome Measures:**

Sexual dysfunction (Female Sexual Function Index; FSFI ≤ 26), Subfertility at 1 and 2 years.

**Results:**

Sexual dysfunction as per the FSFI was more common in patients and associated with poor functional outcomes; sexual abstinence seemed to associate even more so with poor bowel outcomes. Subfertility was higher in patients compared to controls (1 year: 21/45 (47%) vs. 38/178 (21%), *p* = 0.0008; 2 years: 12/45 (27%) vs. 17/178 (10%), *p* = 0.004). There was an increased proportion of patients who had accessed fertility services (20/45 (44%) vs. 43/178 (24%); *p* = 0.009), the proportion of successful pregnancies in patients attempting to conceive with IVF (11/17 (65%) vs. 27/43 (63%); *p* = 1.0) was similar.

**Conclusions:**

These novel data suggest that women with Hirschsprung disease who have undergone reconstructive surgery may be at risk for adverse sexual function and fertility outcomes.

## 1 Introduction

Hirschsprung disease (HSCR) constitutes a congenital absence of ganglion cells within the myenteric and submucosal plexus of a segment of the distal bowel. There is a well‐recognised preponderance in males as well as a known association with certain syndromes, most commonly Trisomy 21, and causative genes, most commonly RET [1, 2, 3]. While the majority (70%–80%) of patients will have disease limited to the rectum and sigmoid colon, a proportion of patients may have extended segment disease, with a loss of the male preponderance (approximately 3.5:1) in extended segment disease, total colonic aganglionosis (TCA) and small intestinal aganglionosis [4]. HSCR usually presents in the neonatal period with signs of lower gastrointestinal obstruction (i.e., abdominal distension and failure to pass meconium), but may present beyond infancy with failure to thrive and a history of constipation, often accompanied by abdominal distension and vomiting.

Management for HSCR is surgical and involves an anastomosis of proximal, ganglionic bowel to the anorectum. There are a variety of techniques described for this, with comparable long‐term functional results [5]. A good outcome, in the form of a bowel function that is within the normal limit, can be expected in approximately half of all adult patients [6, 7, 8, 9], and there is a strong correlation between poor function and a reduced quality of life. There is a paucity of data to describe the long‐term outcome in other functional domains. Recently, a strong focus has been placed on the sexual function and fertility impact that colorectal surgery may have on both men and women, with impaired fertility noted in females after ileoanal pouch surgery following colectomy [10]. Single centre data has suggested a potential impact on sexual function and fertility in females with HSCR, while this appears to be generally preserved in males [11]. Additionally, population‐level data from Sweden has shown that women with HSCR are older at their first child and have fewer children than age‐matched healthy controls [12].

This study sought to obtain detailed functional data from adult women who have undergone surgery for HSCR as children, with the intention of identifying disease‐related factors or other functional domains that might associate with sexual dysfunction. Furthermore, data on fertility outcomes was obtained in order to describe potential issues that might be faced by patients, in comparison to a population of women of similar age from the general population.

## 2 Methods

### 2.1 Study Design

This was a multi‐centre, observational study across four tertiary paediatric surgery centres in different European countries (United Kingdom, Finland, Norway, Sweden). Participants were invited to complete an online survey. Patients completed this with a study participation ID which allowed linkage of responses to medical and surgical records kept at the individual paediatric surgery centre. Data were shared in anonymised format between centres.

### 2.2 Inclusion and Exclusion Criteria

We included patients who were assigned the female sex at birth and had a histology‐confirmed diagnosis of Hirschsprung disease. Patients were identified through institutional coding searches (ICD‐8: 751.39, ICD‐9: 751D, ICD‐10: Q431). As this survey pertained in part to sexual and fertility history, a lower age limit of 20 years was applied; an upper limit of 50 years was applied corresponding to the introduction of the ICD‐8, as patients older than this would not be reliably detected through coding searches. Patients were excluded if they had a diagnosed learning disability (usually as part of an associated syndrome such as Trisomy 21) or if no contact information was available on the medical records. Women from the general population between the ages 20–45 years were recruited as controls, identified through the offices for national statistics of Sweden and Finland or within the UK through Prolific (www.prolific.com) which allows selected advertising to anonymous participants based on disclosed demographic data within the platform. Controls were invited to complete the anonymised questionnaire, having been informed of its nature, and were informed that the survey was designed to explore the relationship between bowel, bladder and sexual function and fertility as part of a larger medical research study.

### 2.3 Survey Design

A complete copy of the survey is available in Data S1. Demographic data collected included relationship status, sexual orientation and gender identity, as well as baseline demographics such as bodyweight, smoking status, educational level and occupation. Medical and surgical, as well as specific gynaecological comorbidities, were documented. Surgery was defined as unrelated to HSCR if it was anything other than revision surgery of the pull‐through (including perineal/anal procedures), formation of an end stoma or continence stoma or a procedure for bowel obstruction. Pelvic inflammatory disease, adhesional disease and hydrosalpinx were pooled together as a common condition; we did not enquire into a history of previous sexually transmitted infections.

Current bowel function was assessed with the Bowel Function Score. This can be completed by anyone in intestinal continuity and the 7‐item questionnaire produces a score out of 20, with a minimum score of 1. Scores ≥ 17 are accepted to be ‘normal’ or ‘good’ bowel function, while scores below 12 are considered to reflect ‘poor’ function [13]. We also defined any patient who required Antegrade Colonic Enema or an end stoma as having had a ‘poor’ outcome as this reflected a need for surgical intervention to achieve social continence. Urinary Incontinence and Lower Urinary Tract Symptoms were assessed with a questionnaire based upon the Danish Prostatic Symptom Score (DanPSS) that has been utilised to assess paediatric and adult patients with Hirschsprung previously [14, 15]. These questionnaires have both been used extensively in all languages required for the completion of this international survey.

To assess sexual function, we used the Female Sexual Function Index (FSFI [16]; validated in English, Finnish, Norwegian and Swedish). The FSFI produces 6 subdomain scores, with a maximum score of 36. Those who have not had sexual activity in the preceding 4 weeks are not able to complete 5 of these subdomains and produce only a ‘Desire’ Subdomain. A score ≤ 26 is deemed to reflect sexual dysfunction [17]. The FSFI allows for imputation with > 50% questions answered, therefore these patients were included on subdomains where this was the case and the overall score calculated by imputation.

A series of closed and white‐space questions were also posed to all women who had ever been or tried to fall pregnant regarding their attempts to conceive and experiences during pregnancy and delivery. These questions were forward and backward translated from English by independent, bilingual members of the study team to assure content validity. We defined subfertility as the inability to conceive despite regular, unprotected intercourse for either 1 or 2 years, and infertility or involuntary childlessness as an inability to conceive biological children by any means (including assisted fertility).

### 2.4 Patient Involvement

As this study was observational and hypothesis generating, rather than interventional, we did not explicitly seek guidance from patient groups regarding the content of the survey that was sent out. However, its content was guided by semi‐structured interviews with a population of 36 women who were surveyed as part of a single‐centre study from one of the participating centres [11].

### 2.5 Statistical Analysis

Comparisons between patient and control populations were performed using descriptive, univariate analyses; continuous/ordinal variables were compared using *T*‐test or Mann–Whitney *U*/Kruskal‐Wallis while categorical data were analysed using Chi‐square. Where possible, a mean difference or odds ratio with an accompanying 95% confidence interval is presented. When multiple subdomains were compared within the FSFI, Dunn's correction was performed. Statistical significance was defined as a *p*‐value less than 0.05.

### 2.6 Ethics Statement

This study received research ethics approval across all countries (UK NHS Health Research Authority: 21/LO/0230, HUS Helsinki: HUS/2751/2020 and HUS/180/2020), Karolinska University Hospital: Ethical Review Authority (2021‐0570‐02). Oslo University Hospital: Regional Ethical Committee (150507) and the Hospital's Data Protection Officer (20/19570).

## 3 Results

### 3.1 Participant Demographics and Disease Characteristics

Ninety female patients responded out of a total of 135 who met the inclusion criteria across the four participating centres, giving a response rate of 67% (presented in Table 1). Patients most commonly had rectosigmoid disease (71%) and had undergone a variety of pull‐through procedures, predominantly Duhamel (52%) or Endorectal pull‐through (ERPT, 32%). Forty‐one patients (46%) had undergone an unplanned laparotomy after their first pull‐through, and of these, 11 patients (12%) had undergone a revision of their pull‐through.

**TABLE 1:** Demographics and comorbidities.

	HSCR (*n* = 90)	Control (*n* = 303)	Mean diff. or [95% CI]; *p*‐value
Age, years [mean (sd)]	31.6 (7.0)	31.8 (6.4)	−0.2 [−1.74–1.34]; *p* = 0.799
BMI [mean (sd)]	25.3 (5.9)	26.6 (8.4)	−1.3 [−3.17–0.57; *p* = 0.171]
			**OR [95% CI); *p*‐value**
*Length of aganglionosis,* *n (%)*			
Rectosigmoid	64 (71%)	—	
Extended (colonic)	12 (13%)	—	
Total colonic aganglionosis	7 (8%)	—	
Small intestinal	3 (3%)	—	
Unknown	4 (4%)	—	
*Pull‐through technique,* *n (%)*			
Duhamel	47 (52%)	—	
ERPT (Yancey‐Soave‐Boley)	32 (36%)	—	
Swenson	4 (4%)	—	
Other/Unknown	7 (8%)	—	
*Current ostomy,* *n (%)*			
ACE	2 (2%)	1 (0.3%)	6.86 [0.61–76.59]; p = 0.132—
Ileostomy/Colostomy	8 (9%)	0	
Education level**			
**University**	**43 (48%)**	**201 (66%)**	**0.46 [0.29–0.75]; *p* = 0.002**
**High School (18)**	**20 (22%)**	**50 (17%)**	—
**Secondary School (16)**	**25 (28%)**	**51 (17%)**	—
**Did not finish**	**2 (2%)**	**1 (0.3%)**	—
*Smoking Status,* *n (%)*			
Current	7 (8%)	33 (11%)	0.69 [0.29–1.62]; *p* = 0.436
Former	27 (30%)	72 (24%)	—
Never	56 (62%)	198 (65%)	—
Comorbidity (non‐HSCR) *n* (%)	38 (42%)	117 (39%)	1.16 [0.72–1.87]; *p* = 0.542
Other surgery (non‐HSCR) *n* (%)	52 (58%)	141 (47%)	1.57 [0.98–2.53]; *p* = 0.072
Previously sexually active	85 (94%)	295 (97%)	0.46 [0.15–1.45]; *p* = 0.185
Current Relationship*	**57 (63%)**	**233 (77%)**	**0.52 [0.31–0.86]; *p* = 0.014**
Sexual debut, years [mean (sd)]	17.0 (2.5)	17.0 (2.8)	0.00 [−0.65–0.65]; *p* = 1.00
Ever been, or tried to become, pregnant	46 (51%)	178 (59%)	0.73 [0.46–1.18]; *p* = 0.226

*Note:* Comparison between HSCR and Control; *p*‐values: * < 0.05, ** < 0.01 (Chi‐squared for attainment of University degree).

The patient cohort was compared to a population of 303 controls. Regarding gender, 2% of participants in both groups (2/90 and 6/303) did not identify as female (*p* = 1.00). Fourteen patients (16%) and 51 controls (17%) identified as non‐heterosexual (*p* = 0.872). There was a significant difference in declared relationship status, with 57 patients (63%) either married or in a relationship at the time of participation compared to 233 controls (77% OR 0.52 [0.31–0.86]; *p* = 0.014). A similar proportion of patients and controls had been sexually active and had been, or tried to become, pregnant. There were no significant differences between patient and control groups regarding age, BMI, smoking status, non‐HSCR medical comorbidity or non‐HSCR related surgery. However, there was a difference in the number of participants who had achieved an undergraduate (or equivalent) qualification (48% vs. 66%; *p* = 0.002).

#### 3.1.1 Gynaecological Comorbidities

Among HSCR patients, there was a higher reported incidence of ovarian and adnexal cysts (OR 5.66 [2.33–13.75]; *p* = 0.0002), endometriosis (OR 4.50 [1.18–17.14]; *p* = 0.03) and pelvic inflammatory and adhesional disease (OR 35.0 [7.9–155]; *p* < 0.0001). No patients or controls disclosed a sexually transmitted infection in the context of pelvic adhesions, which were diagnosed either by ultrasound or at laparoscopy (Table S1).

#### 3.1.2 Bowel and Bladder Function

Regarding bowel function, there were 8 patients (9%) who currently have an end stoma, compared to none among the controls. There were 2 patients and 1 control participant who managed with an ACE stoma for continence. Overall BFS and all seven individual items were significantly lower (suggesting inferior function) in patients vs. controls (Figure S1; *p* < 0.0001 for all). There was no statistical evidence for a difference in urological outcomes of urinary tract infection, lower urinary tract symptoms or urinary incontinence between the patient and control groups (Table S2).

#### 3.1.3 Sexual Function

**FIGURE 1:**
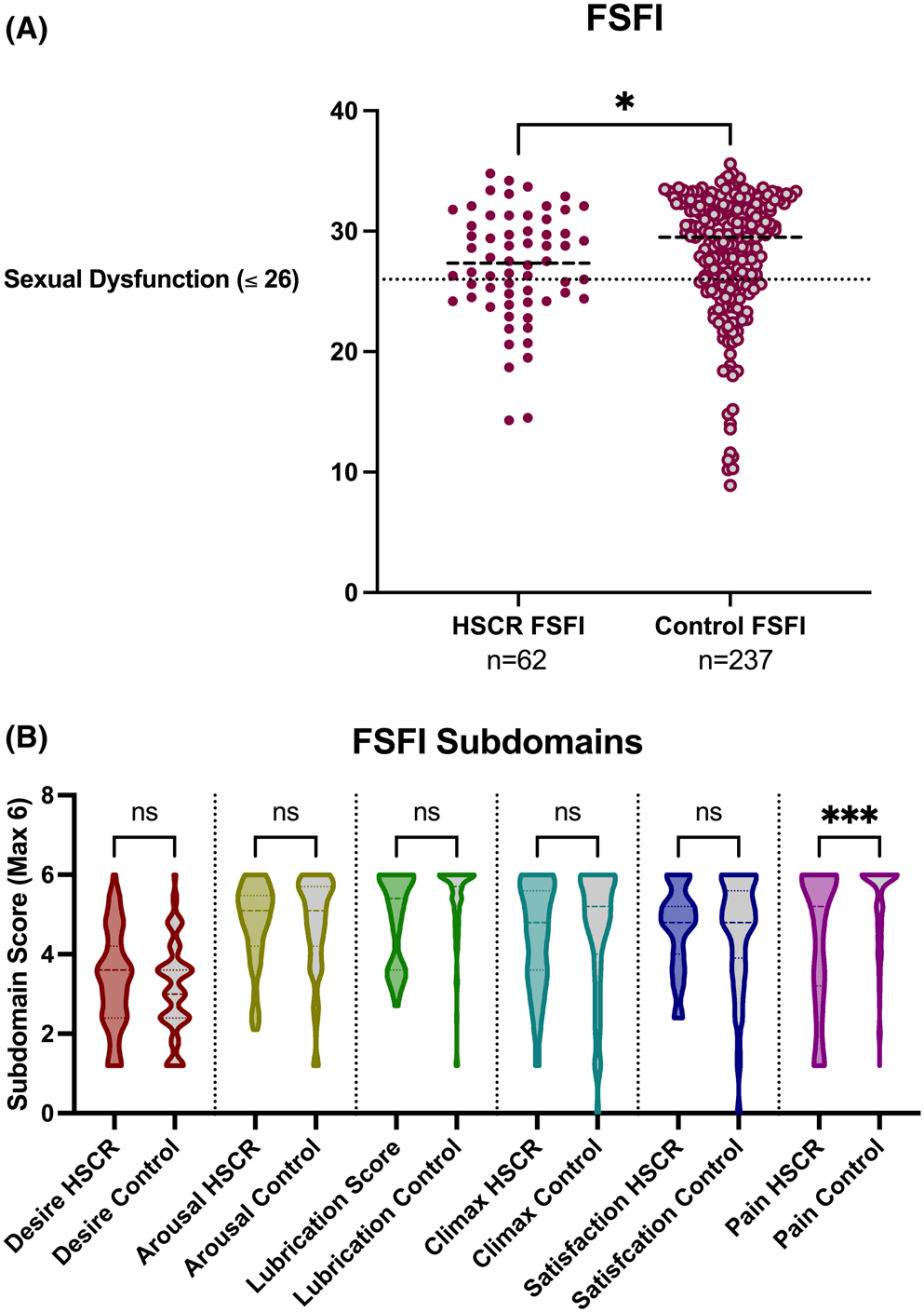
(A) FSFI Scores for HSCR patients and controls. Dashed line for each dataset represents population median and dotted line represents a threshold for sexual dysfunction (FSFI ≤ 26). Comparison with Mann–Whitney *U* test; *p* = 0.035. (B) Comparison of Subdomain scores for patients and controls. Pairwise comparisons with Dunn's test; corrected *p* = 0.0005.

The Female Sexual Function Index (FSFI) was completed by 81/90 (90%) participating patients and 289/303 (95%) controls (OR 0.44 [0.18–1.04]; *p* = 0.07). This index records data across a total of six subdomains, the first of which is ‘Desire’; in the event that a participant had not had any sexual activity in the preceding 4 weeks, their score was limited to this subdomain only. Overall FSFI scores were significantly lower in patients compared to controls (Figure 1A); when subdomain scores were compared, there was no difference between patients and controls for any subdomain except for Pain, which was scored significantly lower (i.e., worse outcome) in patients (Figure 1B). Patients with an overall FSFI score ≤ 26/36 are deemed to have sexual dysfunction, although the proportion of individuals meeting this definition was higher in patients, this was not statistically significant (25/62 (40%) vs. 65/237 (27%), OR 1.79 [1.00–3.20]; *p* = 0.062).

#### 3.1.4 Fertility Outcomes

**FIGURE 2:**
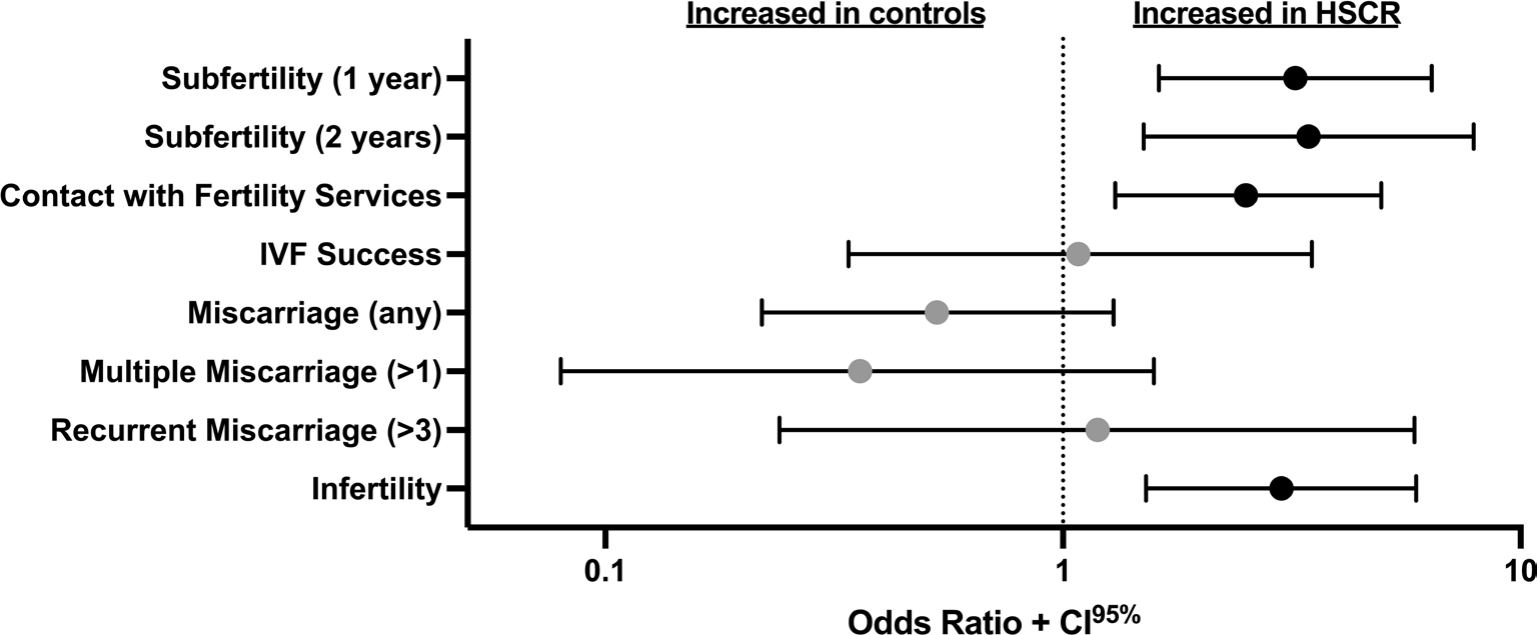
Univariate analysis of fertility outcomes compared between patients and controls. Odds Ratio [95% confidence interval] shown. Significant differences shown by black markers (vs. grey).

Half of HSCR patients, 45/90 (50%), and 178/303 controls (59%; OR 0.70 [0.44–1.13]; *p* = 0.148) had either been pregnant or ever tried to become pregnant, and fertility outcomes were compared between groups (Figure 2). Subfertility was higher in patients compared to controls (at 1 year: 21/45 (47%) vs. 38/178 (21%), OR 3.22 [1.62–6.41] *p* = 0.0008; and at 2 years: 12/45 (27%) vs. 17/178 (10%), OR 3.44 [1.50–7.89] *p* = 0.004). There was no difference in the age at which this subfertility was noted (mean [sd] 26.8 [5.1] years and 27.7 [4.8] years in patients and controls respectively; mean difference −0.9 [−2.5–0.7] years *p* = 0.268). A single miscarriage was reported in 7/35 (20%) patients and in 53/165 (32%) controls who had fallen pregnant (OR 0.53 [0.22–1.29]; *p* = 0.222), whereas multiple miscarriage (> 1) occurred in 2/35 (6%) patients vs. 24/165 (15%) controls; (OR 0.36 [0.08–1.58]; *p* = 0.182) and recurrent miscarriage (≥ 3) in 2/35 (6%) patients vs. 8/165 (5%) controls; (OR 1.19 [0.24–5.86]; *p* = 1.00); but the numbers of individuals with these outcomes were too low to reach any conclusion.

There was an increased proportion of patients who had accessed fertility services (20/45 (44%) vs. 43/178 (24%); OR 2.51 [1.27–4.96]; *p* = 0.009). The proportion of successful pregnancies in patients attempting to conceive with IVF (11/17 (65%) vs. 27/43 (63%); OR 1.09 [0.34–3.50]; *p* = 1.0) were similar between the groups, although the wide confidence intervals make it difficult to reach a conclusion on whether IVF success is truly equivalent. Overall, involuntary childlessness was more common in patients (22/45 (49%) vs. 43/178 (24%); OR 3.00 [1.52–5.91]; *p* = 0.002). Two controls and one patient had adopted.

#### 3.1.5 Pregnancy Outcomes

There was no statistical difference between the age of patients (*n* = 35) and controls (*n* = 164) at the first pregnancy (26.1 (7.1) vs. 25.1 (5.5) years; mean diff. 1.00 [−1.13–3.13]; *p* = 0.356). There was an increased use of Elective Caesarean Section across all pregnancies in the cohort (9/77 (12%) vs. 12/429 (3%); OR 4.60 [1.87–11.33]; *p* = 0.002). Overall, there were fewer live births per pregnancy in the patient group (40/77 (52%) vs. 277/429 (65%); OR 0.59 [0.36–0.97]; *p* = 0.041). Eight patients (out of 35 falling pregnant; 22%) had unplanned admissions to hospital during pregnancy, two of whom had major complications related to bowel adhesions requiring major abdominal surgery and/or expedited delivery of their baby.

Bowel symptomatology changed variably during pregnancy, with almost one third of patients (10/35; 29%) experiencing some change. Five referred to worsening constipation, while three mentioned worse bloating with diarrhoea. Interestingly, two patients stated that their symptoms of bloating and diarrhoea improved. Constipation during pregnancy was common among controls, declared by 40/165 women (24%). Vaginal delivery (compared to giving birth by caesarean section) was associated with urinary incontinence (OR 7.79 [1.77–34.26]; *p* = 0.003) and faecal soiling (OR 3.06 [1.19–7.89]; *p* = 0.027) in controls, but the number of patients experiencing these outcomes in the HSCR patient group was too low to reach any conclusion (Faecal soiling OR 1.46 [0.34–6.35]; *p* = 0.714. Urinary incontinence (OR 0.52 [0.09–3.14]; *p* = 0.679); Table S3).

#### 3.1.6 Predictors of Poor Sexual Function in Patients

**FIGURE 3:**
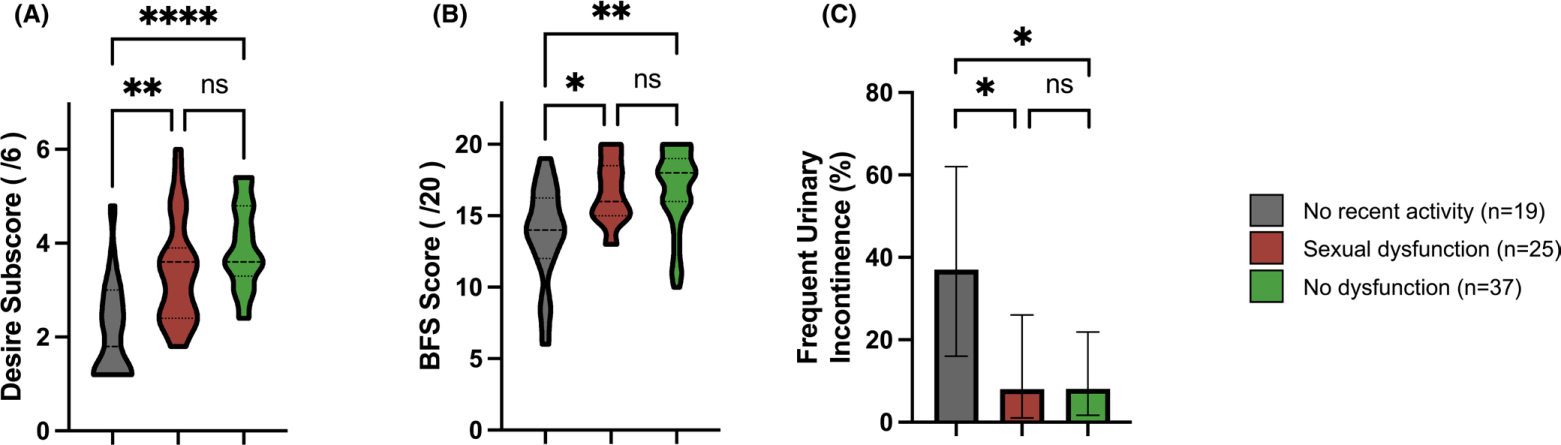
Comparison of patients with recent sexual abstinence vs. FSFI defined sexual dysfunction vs. no sexual dysfunction. (A) Sexual Desire FSFI Subdomain Scores, (B) Bowel Function Scores, (C) Prevalence of frequent (i.e., weekly) urinary incontinence. Comparison with Kruskal‐Wallis and Dunn's test for pairwise comparison. Corrected *p*‐values; *p < 0.05, **p < 0.01, ****p < 0.0001.

Assessing those who had not had recent sexual activity and therefore did not complete the entire FSFI (*n* = 19), this specific group of patients had a lower Desire sub‐score than either patients with sexual dysfunction or those without, and also had a lower Bowel function Score and a higher incidence of frequent urinary incontinence (i.e. more than weekly episodes) (Figure 3).

## 4 Discussion

### 4.1 Main Findings

This study is the first to describe detailed sexual function and fertility outcomes across a cohort of women with Hirschsprung disease. Comparison of patient‐derived data with control data from the general population identifies a number of significant differences that merit further study and should inform the wider care of adolescent and adult patients with HSCR regarding their family planning and management by specialist gynaecological and fertility services. Pelvic adhesive disease was more than thirty times more prevalent among patients, a condition that must be assumed to be related to the pull‐through surgery they have undergone as children. There was also an increased incidence of endometriosis among patients, which could be related to mechanical obstruction of menses as a result of the aforementioned adhesions. The increased incidence of ovarian cysts may be more related to an increased proportion of women who may be undergoing imaging investigations for other reasons such as gastrointestinal symptoms.

Pelvic adhesions are known to be a common occurrence after colorectal surgery in adults and have well‐recognised morbidity, including sexual dysfunction and infertility [10, 18, 19]. This has led to a proposed option to defer definitive reconstructive ileal‐pouch surgery after colectomy in women until after child‐bearing age [20]. The potential relevance of pelvic adhesions was highlighted by the fact that, overall, HSCR patients appeared to have poorer sexual function (manifested in lower FSFI scores with a lower Pain subscore) and that their fertility is significantly impacted. This has also been recognised as a prevalent complication of pelvic colorectal surgery in women [21]. The adverse fertility outcomes among female HSCR patients are a novel finding, previously suggested by a small single‐centre cohort from within this research group [11]. On a population level, women with HSCR have also been shown to have fewer children and be older at the time of having their first child [12]. Other unrecognised factors that may affect fertility in these women could also be considered; for instance, it is unknown whether RET or other mutations causing HSCR might affect fertility independently of surgery or its sequelae. For example, to our knowledge, there has been no study of motility in the fallopian tubes of women with Hirschsprung.

We observed that women with Hirschsprung who had sexual dysfunction also appeared to have worse bladder and bowel outcomes. It is therefore important to recognise the complex interplay between sexual function, quality of life and bowel and bladder continence, which are known to cluster together in individuals with HSCR [5, 6, 8, 14]. We have previously advocated that patients with any identifiable impairment should undergo multi‐disciplinary transitional care in order to assure that physical and psychological support is provided where needed [22, 23]. Importantly, however, we were not able to identify any disease‐related factors or outcomes that associated with poor fertility in our patient cohort, suggesting that all patients should be specifically counselled about this matter.

### 4.2 Strengths and Limitations

This study has several limitations, largely related to the patient‐reported nature of outcomes and the lack of access to adult medical records in order to validate self‐declared conditions such as pelvic adhesional disease. The patient‐reported nature of the data will mean that there is a susceptibility to selection bias within the data, with patients with higher symptom burden more likely to want to share their story and report their outcomes—it is difficult to know if this would be similar among controls. Furthermore, patients with a prior medical history may be more astute and cognisant of symptoms and therefore more likely to undergo investigations, receive and recall diagnoses for symptoms.

With follow‐up approaching 70%, the results observed should be recognised as relevant, but the possibility of a Type I error when so many comparisons are made between groups remains. The most important findings we have described—those of subfertility and involuntary childlessness—were observed with sufficient difference from the control population that the risk of false positive should be regarded as low. In addition, interpretation of some fertility outcomes, such as the comparison of live births per pregnancy between patients and controls, is complicated by the finding that patients were significantly less likely to achieve pregnancy than controls. Patient‐level data were not available for all eligible patients in all centres; therefore, it was not possible to perform a comparison of patient and disease factors between those who took part and those who were not successfully contacted or declined to participate.

Regarding attrition bias, a proportion of participants (10% of patients, 5% of controls) opted not to complete the FSFI and therefore there remains an uncertainty as to the sexual functional outcomes in a proportion of participants. Clearly, disclosing information regarding sexuality, even in an anonymised survey, is highly sensitive, and it is uncertain how those non‐respondents may have influenced the overall results. Given our observation that those patients who had the most severe functional outcome also had lower sexual desire subscores and more commonly had been sexually abstinent, it might be assumed that those patients declining to complete this aspect of the study questionnaire may be similarly affected. Although this study represents, by far, the largest study of adult female HSCR patients in the literature, the total patient numbers may still preclude statistical confirmation of associations with rare events (i.e. infertility and redo surgery are both reported in only a proportion of patients). We believe the results presented here are supported by the inclusion of population controls of similar age and nationality, which aids the contextualisation of the patient data. With more robust quantitative data and prospectively collected outcomes according to agreed criteria, it might be possible to further assess this particular group of HSCR patients with regard to the complex interplay of functional and psychosocial factors that may affect both sexual function and fertility, in order to best support them as they move through adolescence and young adulthood.

### 4.3 Interpretation

These data suggest an association of HSCR surgery in women with impaired fertility and sexual function. Whether these observations may change after the introduction of minimally invasive techniques for pull‐through surgery in the last two decades remains to be seen, as those patients will only just be reaching childbearing age now, reinforcing the need to continue to study this important area in the future. Inferences from the literature for ileo‐anal pouch surgery in adults are encouraging [24, 25], these may not apply to HSCR given the age and anatomy of the infant pelvis (most pull‐through surgery performed prior to 1 year of age).

## 5 Conclusion

These data provide a first look at patient‐level data related to sexual function and fertility in women after surgery for HSCR. Factors associated with poor outcome are explored, and importantly there does not appear to be an identifiable risk factor for the impaired fertility which iscommonly identified in this patient group. The responsibility for imparting this advice should remain with the paediatric treatment team and the conversation should begin during the process of transitioning care to adult services. We feel the information here should empower patients with knowledge relevant to their family planning and future fertility journey. We believe that supportive advice regarding family planning should be offered to all adolescent female patients with Hirschsprung, with advice to seek help early if they encounter difficulties conceiving, and we hope that these data are able to raise awareness among obstetricians and gynaecologists who may encounter women affected by this condition.

## Author Contributions

J.R.D. designed the study, collected and analysed data and wrote the initial manuscript. A.M., A.L.G. and A.T.H. co‐designed the study, collected data and wrote the initial manuscript. S.E. supervised the study design, analysed the data and provided critical review of the manuscript. S.P.L., P.D.C., K.B., T.W., M.P.P., and J.C. provided supervisory support for all stages of the study (design, data collection, analysis and presentation) and provided critical review of the manuscript. All authors have approved the manuscript in its submitted form.

## Disclosure

Data from this study has been presented at the British Association of Paediatric Surgeons (BAPS) Annual Congress 2023, the European Paediatric Surgery Association (EUPSA) Annual Congress 2023, the StayCurrentMD Best of the Best in Paediatric Surgery 2024 and the Paediatric Colorectal Club 2024.

## Ethics Statement

This study received research ethics approval across all countries in March–April 2021: (UK NHS Health Research Authority: 21/LO/0230, HUS Helsinki: HUS/2751/2020 and HUS/180/2020), Karolinska University Hospital: Ethical Review Authority (2021‐0570‐02). Oslo University Hospital: Regional Ethical Committee (150507) and the Hospital's Data Protection Officer (20/19570).

## Conflicts of Interest

The authors declare no conflicts of interest.

## Supporting information


**Table S1.** Gynaecological Comorbidities; comparison using Fisher’s Exact with accompanying odds ratio + 95% confidence interval.
**Figure S1.** Bowel function score for HSCR patients vs. controls. Scores ≥ 17 considered ‘Normal’ and < 12 considered poor. Comparison with Mann–Whitney *U* test; *p* < 0.0001.
**Table S2.** Urological outcomes comparing HSCR patients and controls, data presented as *n* (%). Statistical comparison with Fisher’s exact.
**Table S3.** Bowel and bladder impairment in patients and controls after live birth delivery, comparison between women who had given birth vaginally and those who had been pregnant but not delivered vaginally. *Urinary incontinence outcome includes 2 HSCR patients with colostomy (i.e., not suitable for bowel outcome assessment) who had not had a vaginal delivery.

**Data S1.** GOSH patient questionnaire.

## Data Availability

Anonymised control data are available upon reasonable request. Patient data require further ethical approval to share beyond the use in this study.
