# Supplementary material for: Impaired Fertility and Sexual Function in Women With Hirschsprung Disease: Results From an International Multi‐Centre Cross‐Sectional Study
Source: BJOG. 2025 Jul 10;132(11):1673–80. doi: 10.1111/1471-0528.18294 (PMC12411651; doi:10.1111/1471-0528.18294)
Supplement: Supplementary file 1 — Table S1. Gynaecological Comorbidities; comparison using Fisher’s Exact with accompanying odds ratio + 95% confidence interval. Figure S1. Bowel function score for HSCR patients vs. controls. Scores ≥ 17 considered ‘Normal’ and < 12 considered poor. Comparison with Mann–Whitney U test; p < 0.0001. Table S2. Urological outcomes comparing HSCR patients and controls, data presented as n (%). Statistical comparison with Fisher’s exact. Table S3. Bowel and bladder impairment in patients and controls after live birth delivery, comparison between women who had given birth vaginally and those who had been pregnant but not delivered vaginally. *Urinary incontinence outcome includes 2 HSCR patients with colostomy (i.e., not suitable for bowel outcome assessment) who had not had a vaginal delivery. [file BJO-132-1673-s002.docx]

**Table S1. Gynaecological Comorbidities; comparison using Fisher’s Exact with accompanying Odds Ratio + 95% Confidence Interval**

| **Diagnosis** | **HSCR**  **(n=90)** | **Control**  **(n=303)** | **OR + CI^95%^ ;**  **p-value** |
| --- | --- | --- | --- |
| Polycystic Ovarian Syndrome | 3 (3.3%) | 26 (8.6%) | 0.38 [0.11-1.27]; p=0.11 |
| **Ovarian/Adnexal Cyst** | **13 (14%)** | **9 (3%)** | **5.66 [2.33-13.75]; p=0.0002** |
| **Endometriosis** | **5 (5.6%)** | **4 (1.3%)** | **4.50 [1.18-17.14]; p=0.03** |
| Fibroid/Adenomyosis | 1 (1.1%) | 8 (2.6%) | 0.42 [0.05-3.43]; p=0.48 |
| CIN / Cervical HPV | 2 (2.2%) | 19 (6.3%) | 0.35 [0.08-1.52]; p=0.18 |
| Pelvic Prolapse | - | 2 (0.7%) | n/a; p=1 |
| **Pelvic Inflammatory Disease / Hydrosalpinx** | **17 (19%)** | **2 (0.7%)** | **35.0 [7.9-155]; p<0.0001** |

**Figure S1. Bowel Function Score for HSCR Patients vs Controls. Scores ≥17 considered “Normal” and <12 Considered Poor. Comparison with Mann-Whitney U Test; p<0.0001.**

**Table S2. Urological Outcomes comparing HSCR patients and controls, data presented as n(%). Statistical comparison with Fisher’s Exact**

| Outcome | HSCR (n=90) | Controls (n=303) | OR + CI^95%^ ; p-value |
| --- | --- | --- | --- |
| Urinary Tract Infection  History of UTI  UTI in past 12 months | 61 (68)  23 (25) | 189 (62)  62 (20) | 1.27 [0.77-2.09]; p=0.384  1.33 [0.77-2.31]; p=0.310 |
| Lower Urinary Tract Symptoms  Urinary Frequency (>8/day)  Urinary Urgency | 13 (14)  46 (51) | 50 (17)  155 (51) | 0.854 [0.44-1.66]; p=0.744  1.00 [0.62-1.60]; p=1.00 |
| Urinary Incontinence  Urge Incontinence (Any)  Weekly Urge Incontinence  Stress Incontinence (Any)  Weekly Stress Incontinence | 28 (31)  5 (6)  42 (47)  11 (12) | 69 (23)  8 (3)  153 (50)  33(11) | 1.53 [0.91-2.58]; p=0.125  2.17 [0.69-6.80]; p=0.185  0.86 [0.54-1.37]; p=0.550  1.14 [0.55-2.36]; p=0.849 |
| Psychological or Social Issues | 5 (6) | 15 (5) | 1.13 [0.40-3.20]; p=1.00 |

**Table S3. Bowel and Bladder Impairment in patients and controls after live birth delivery, comparison between women who had given birth vaginally and those who had been pregnant but not delivered vaginally. *Urinary incontinence outcome includes 2 HSCR patients with colostomy (i.e. not suitable for bowel outcome assessment) who had not had a vaginal delivery.**

|  | **HSCR (n=33)** | | | **Controls (n=165)** | | |
| --- | --- | --- | --- | --- | --- | --- |
|  | **Vaginal Delivery (n=13)** | **No Vaginal Delivery (n=20)** | **OR [CI 95%; p =)** | **Vaginal Delivery (n=110)** | **No Vaginal Delivery (n=55)** | **OR [CI 95%; p =)** |
| **Faecal Soiling** | 5 (38%) | 6 (30%) | 1.46 [0.34 – 6.35]; p=0.714 | 30 (27%) | 6 (11%) | 3.06 [1.190-7.89]; p=0.027 |
| **Faecal Accidents** | 3 (23%) | 5 (25%) | 0.90 [0.17-4.64]; p=1.0 | 12 (11%) | 3 (4%) | 2.12 [0.57-7.86]; p=0.390 |
| **Frequent Urinary Incontinence*** | 2 (15%) | 5 (23%) | 0.52 [0.09-3.14]; p=0.679 | 25 (23%) | 2 (4%) | 7.79 [1.77-34.26]; p=0.003 |
