# Supplementary material for: Impaired Fertility and Sexual Function in Women With Hirschsprung Disease: Results From an International Multi‐Centre Cross‐Sectional Study
Source: BJOG. 2025 Jul 10;132(11):1673–80. doi: 10.1111/1471-0528.18294 (PMC12411651; doi:10.1111/1471-0528.18294)
Supplement: Supplementary file 2 — Data S1. GOSH patient questionnaire. [file BJO-132-1673-s001.pdf]

GOSH Patient Questionnaire

Thank you for agreeing to take part in this questionnaire, the following quesitons will cover your general health and medical history, before asking specifically about your bowel and urinary function and then finally asking about your history regarding sex and fertility. Please remember you are under no obligation to complete the questionnaire and can stop at any time. If there are any questions you have about the questionnaire or your responses to it, please contact one of the study team

Please enter your study ID, which will have been given to you on your consent form by a member of the study team. ______________________

What is your current height (in cm)? ______________________

What is your current weight (in kg)? ______________________

What is your current age (in years)? ______________________

To which gender do you most identify:
- ◯ Female
- ◯ Male
- ◯ Transgender female
- ◯ Transgender male
- ◯ Non-binary / Gender Queer / Gender Fluid
- ◯ Don't know
- ◯ Prefer not to say
- ◯ Other (not listed here)

Please specify: ______________________

How would you describe your sexual orientation
- ◯ Heterosexual (attracted to opposite sex)
- ◯ Homosexual (attracted to same sex)
- ◯ Bisexual (attracted to both sexes)
- ◯ Don't know
- ◯ Prefer not to say
- ◯ Other

Please specify: ______________________

What is your current relationship status?
- ◯ Married / Civil Partnership
- ◯ Divorced
- ◯ In a relationship
- ◯ Single
- ◯ Prefer not to answer
- ◯ Other

Please specify:	__________________________
Have you engaged in sexual intercourse?	◯ Yes<br>◯ No
At what age did this first happen (in years)	__________________________<br>(If you would prefer not to disclose this please leave blank)
What is your current education level?	◯ Did not complete GCSE / 16+ Exams<br>◯ Completed GCSE / 16+ Exams (i.e. NVQ or BTEC Level 1 or 2)<br>◯ Completed A-Levels / NVQ or BTEC Level 3<br>◯ Completed University Degree / Higher Education Qualification (i.e. NVQ or BTEC Level 4 / 5)<br>(Please note your highest completed level of education only)
Please give any additional information about your education that may be useful:	________________________________
What is your occupation?	__________________________
Are you a smoker?	◯ Never smoked<br>◯ Used to smoke<br>◯ Currently smoke
How many years did you smoke for?	__________________________
At what age did you start smoking?	__________________________
How many cigarettes per day?	◯ Less than 1 per day<br>◯ 1-5<br>◯ 6-10<br>◯ 11-20<br>◯ More than 20
Other than your Hirschsprung's, have you been diagnosed with any other medical conditions?	◯ Yes<br>◯ No
Could you specify what these are?	________________________________
Other than surgery for your Hirschsprung's have you have any other surgical procedures?	◯ Yes<br>◯ No
Could you specify what these are?	________________________________

Have you been diagnosed with any gynaecological conditions (these are conditions affecting the ovaries, fallopian tube, uterus or vagina)?	◯ Yes<br>◯ No
Could you specify what these are?	__________________________
Have you been diagnosed with any hormonal conditions (these might include thyroid / diabetes / polycystic ovaries)?	◯ Yes<br>◯ No
Could you specify what these are?	__________________________
Please list any regular medication you take:	__________________________<br>(Please write none, if you do not take any)

We want to ask about your bowel and bladder function, we are interested to explore how this might affect sex and fertility

Do you currently have a stoma (colostomy or ileostomy) ?	◯ Yes<br>◯ No
Do you currently use an ACE (appendicostomy for antegrade colonic enema) ?	◯ Yes<br>◯ No
You are aware of the feeling when you need to pass stool	◯ Always<br>◯ Most of the time<br>◯ Uncertain<br>◯ Never
You are able to hold back passing stool	◯ Always<br>◯ Problems less than once per week<br>◯ Weekly problems<br>◯ No voluntary control
You typically pass stool	◯ Less than once every 2 days<br>◯ Once every 2 days<br>◯ Once a day<br>◯ Twice a day<br>◯ More than twice a day
How many times do you need to pass stool on an average day?	_____________________
Do you have problems with faecal soiling (staining of the underwear)	◯ Never<br>◯ Problems less than once per week, change of underwear only needed rarely<br>◯ Weekly problems, change of underwear often needed<br>◯ Daily problems, requiring protective aids (i.e. pads or diapers)

Do you have accidents involving stool	◯ Never<br>◯ Less than once per week<br>◯ Weekly accidents, requiring protective aids<br>◯ Daily problems, requiring protective aids day and night
Do you suffer from constipation?	◯ No constipation<br>◯ Constipation managed with diet alone<br>◯ Constipation managed with medication<br>◯ Constipation managed with enemas
Does your bowel function affect your social life?	◯ No impact<br>◯ Some impact (i.e. bad smells sometimes)<br>◯ Problems that restrict social activities<br>◯ Major social or psychological problems as a result of bowel function
Please use this space to tell use anything more about your bowel habits	________________________________
Have you ever had a urinary tract infection (UTI) ?	◯ Yes<br>◯ No
How many UTI have you had in the past year?	__________________________
How many times do you pass urine each day?	◯ 1 - 3 times a day<br>◯ 4 - 8 times a day<br>◯ More than 8 times a day
Do you ever need to strain to start/continue urination?	◯ Never<br>◯ Rarely (less than once per week)<br>◯ Often (more than once per week)<br>◯ Always
Do you ever get a sudden urge to pass urine?	◯ Never<br>◯ Rarely (less than once per week)<br>◯ Often (more than once per week)<br>◯ Always
Is the urge so strong that urine escapes before reaching the toilet?	◯ Never<br>◯ Rarely (less than once per week)<br>◯ Often (more than once per week)<br>◯ Always
Does urine ever leak upon straining (such as laughing, sneezing or coughing)?	◯ Never<br>◯ Rarely (less than once per week)<br>◯ Often (more than once per week)<br>◯ Always
Does urine ever leak without physical activity or apparent need to urinate?	◯ Never<br>◯ Rarely (less than once per week)<br>◯ Often (more than once per week)<br>◯ Always

Do you ever have night-time wetting (bed wetting)?	◯ Never<br>◯ Rarely (less than once per week)<br>◯ Often (more than once per week)<br>◯ Always
Do you consider yourself to have social problems as a result of urinary incontinence?	◯ No<br>◯ Yes, due to daytime urinary incontinence only<br>◯ Yes, due to night-time urinary incontinence only<br>◯ Yes, due to both daytime and night-time urinary incontinence
Please use this space to tell use anything more about your urinary function	__________________________

Thank you for completing the questionnaire so far, the next sections will ask about your menstrual history and sexual history

At what age (in years) did you have your first menstrual period?	______________________<br>(If you have not had a period please note this in the box)
Does your menstrual period almost always come about monthly?	◯ Yes<br>◯ No
Is your menstrual period usually the same duration and heaviness each time?	◯ Yes<br>◯ No
What is the usual number of periods you might expect in one year?	◯ 0 - 1<br>◯ 2 - 4<br>◯ 5 - 7<br>◯ 8 - 10<br>◯ 11 or more
How many in one year?	______________________
Your period flow is	◯ Often different one period to the next<br>◯ Very Light<br>◯ About Normal<br>◯ Very Heavy<br>◯ Other
Could you tell us more?	__________________________
The duration of your period is	◯ Often different one period to the next<br>◯ Less than 5 days<br>◯ 5 - 10 days<br>◯ More than 10 days
Have you ever used contraception (including for managing your periods)?	◯ Yes<br>◯ No

Please tell us which methods you have used (select all that apply)	☐ Condoms<br>☐ Oral Contraceptive Pill (Combined or Progesterone Only)<br>☐ Long-acting Hormonal (e.g. Injection, Implant, IUS [i.e. Mirena Coil])<br>☐ Male partner vasectomy<br>☐ Tubal ligation<br>☐ Other (please specify)
Please tell us which other type of contraception you have used?	__________________________________
Do you currently use contraception?	◯ Yes<br>◯ No
Please tell us which methods you currently use (select all that apply)	☐ Condoms<br>☐ Oral Contraceptive Pill (Combined or Progesterone Only)<br>☐ Long-acting Hormonal (e.g. Injection, Implant, IUS [i.e. Mirena Coil])<br>☐ Male partner vasectomy<br>☐ Tubal ligation<br>☐ Other (please specify)
Please tell us which other type of contraception you currently use?	__________________________________
Have you ever been pregnant?	◯ Yes<br>◯ No
How many times have you ever been pregnant, counting all pregnancies at all times in your life, regardless of the outcome?	__________________________________
During your pregnancy did you notice any change to the symptoms of your Hirschsprung's?	◯ Yes<br>◯ No
Could you tell us more about what happened?	____
Did you have any unplanned admissions to hospital during your pregnancy?	◯ Yes<br>◯ No
Could you tell us more about what happened?	____
How many pregnancies resulted from attempts to conceive?	__________________________________
How many pregnancies resulted when you were using a method to prevent pregnancy?	__________________________________
Could you give us some more information about your pregnancy?	
Could you give us some more information about your [pregnancy_number] pregnancies	

How old were you at your first pregnancy (years)?	__________________________
What was the outcome of this pregnancy?	◯ Live birth<br>◯ Still birth<br>◯ Miscarriage<br>◯ Termination of pregnancy<br>◯ Other (please specify)
Please tell us how the pregnancy ended?	__________________________
How did you deliver?	◯ Vaginal delivery<br>◯ Instrument delivery (i.e. forceps or suction)<br>◯ Elective Caesarean Section<br>◯ Emergency Caesarean Section<br>◯ Other (please specify)
Please tell us about the delivery in this pregnancy	__________________________
At how many weeks gestation?	__________________________
Could you tell us any more about the pregnancy (i.e. twins, did you use assisted fertility etc.)	________________________________
How old were you at your second pregnancy (years)?	__________________________
What was the outcome of this pregnancy?	◯ Live birth<br>◯ Still birth<br>◯ Miscarriage<br>◯ Termination of pregnancy<br>◯ Other (please specify)
Please tell us how the pregnancy ended?	__________________________
How did you deliver?	◯ Vaginal delivery<br>◯ Instrument delivery (i.e. forceps or suction)<br>◯ Elective Caesarean Section<br>◯ Emergency Caesarean Section<br>◯ Other (please specify)
Please tell us about the delivery in this pregnancy	__________________________
At how many weeks gestation?	__________________________
Could you tell us any more about the pregnancy (i.e. twins, did you use assisted fertility etc.)	________________________________

How old were you at your 3rd pregnancy (years)?	______________________
What was the outcome of this pregnancy?	◯ Live birth<br>◯ Still birth<br>◯ Miscarriage<br>◯ Termination of pregnancy<br>◯ Other (please specify)
Please tell us how the pregnancy ended?	______________________
How did you deliver?	◯ Vaginal delivery<br>◯ Instrument delivery (i.e. forceps or suction)<br>◯ Elective Caesarean Section<br>◯ Emergency Caesarean Section<br>◯ Other (please specify)
Please tell us about the delivery in this pregnancy	______________________
At how many weeks gestation?	______________________
Could you tell us any more about the pregnancy (i.e. twins, did you use assisted fertility etc.)	____________________________
How old were you at your 4th pregnancy (years)?	______________________
What was the outcome of this pregnancy?	◯ Live birth<br>◯ Still birth<br>◯ Miscarriage<br>◯ Termination of pregnancy<br>◯ Other (please specify)
Please tell us how the pregnancy ended?	______________________
How did you deliver?	◯ Vaginal delivery<br>◯ Instrument delivery (i.e. forceps or suction)<br>◯ Elective Caesarean Section<br>◯ Emergency Caesarean Section<br>◯ Other (please specify)
Please tell us about the delivery in this pregnancy	______________________
At how many weeks gestation?	______________________
Could you tell us any more about the pregnancy (i.e. twins, did you use assisted fertility etc.)	____________________________

How old were you at your 5th pregnancy (years)? ______________________________

What was the outcome of this pregnancy?
- ◯ Live birth
- ◯ Still birth
- ◯ Miscarriage
- ◯ Termination of pregnancy
- ◯ Other (please specify)

Please tell us how the pregnancy ended? ______________________________

How did you deliver?
- ◯ Vaginal delivery
- ◯ Instrument delivery (i.e. forceps or suction)
- ◯ Elective Caesarean Section
- ◯ Emergency Caesarean Section
- ◯ Other (please specify)

Please tell us about the delivery in this pregnancy ______________________________

At how many weeks gestation? ______________________________

Could you tell us any more about the pregnancy (i.e. twins, did you use assisted fertility etc.) ______________________________________

How old were you at your 6th pregnancy (years)? ______________________________

What was the outcome of this pregnancy?
- ◯ Live birth
- ◯ Still birth
- ◯ Miscarriage
- ◯ Termination of pregnancy
- ◯ Other (please specify)

Please tell us how the pregnancy ended? ______________________________

How did you deliver?
- ◯ Vaginal delivery
- ◯ Instrument delivery (i.e. forceps or suction)
- ◯ Elective Caesarean Section
- ◯ Emergency Caesarean Section
- ◯ Other (please specify)

Please tell us about the delivery in this pregnancy ______________________________

At how many weeks gestation? ______________________________

Could you tell us any more about the pregnancy (i.e. twins, did you use assisted fertility etc.) ______________________________________

How old were you at your 7th pregnancy (years)? ________________________

What was the outcome of this pregnancy?
- ◯ Live birth
- ◯ Still birth
- ◯ Miscarriage
- ◯ Termination of pregnancy
- ◯ Other (please specify)

Please tell us how the pregnancy ended? ________________________

How did you deliver?
- ◯ Vaginal delivery
- ◯ Instrument delivery (i.e. forceps or suction)
- ◯ Elective Caesarean Section
- ◯ Emergency Caesarean Section
- ◯ Other (please specify)

Please tell us about the delivery in this pregnancy ________________________

At how many weeks gestation? ________________________

Could you tell us any more about the pregnancy (i.e. twins, did you use assisted fertility etc.) ______________________________

How old were you at your 8th pregnancy (years)? ________________________

What was the outcome of this pregnancy?
- ◯ Live birth
- ◯ Still birth
- ◯ Miscarriage
- ◯ Termination of pregnancy
- ◯ Other (please specify)

Please tell us how the pregnancy ended? ________________________

How did you deliver?
- ◯ Vaginal delivery
- ◯ Instrument delivery (i.e. forceps or suction)
- ◯ Elective Caesarean Section
- ◯ Emergency Caesarean Section
- ◯ Other (please specify)

Please tell us about the delivery in this pregnancy ________________________

At how many weeks gestation? ________________________

Could you tell us any more about the pregnancy (i.e. twins, did you use assisted fertility etc.) ______________________________

How old were you at your 9th pregnancy (years)?

____

What was the outcome of this pregnancy?

- ◯ Live birth
- ◯ Still birth
- ◯ Miscarriage
- ◯ Termination of pregnancy
- ◯ Other (please specify)

Please tell us how the pregnancy ended?

____

How did you deliver?

- ◯ Vaginal delivery
- ◯ Instrument delivery (i.e. forceps or suction)
- ◯ Elective Caesarean Section
- ◯ Emergency Caesarean Section
- ◯ Other (please specify)

Please tell us about the delivery in this pregnancy

____

At how many weeks gestation?

____

Could you tell us any more about the pregnancy (i.e. twins, did you use assisted fertility etc.)

____

How old were you at your 10th pregnancy (years)?

____

What was the outcome of this pregnancy?

- ◯ Live birth
- ◯ Still birth
- ◯ Miscarriage
- ◯ Termination of pregnancy
- ◯ Other (please specify)

Please tell us how the pregnancy ended?

____

How did you deliver?

- ◯ Vaginal delivery
- ◯ Instrument delivery (i.e. forceps or suction)
- ◯ Elective Caesarean Section
- ◯ Emergency Caesarean Section
- ◯ Other (please specify)

Please tell us about the delivery in this pregnancy

____

At how many weeks gestation?

____

Could you tell us any more about the pregnancy (i.e. twins, did you use assisted fertility etc.)

____

How old were you at your 11th pregnancy (years)?	__________________
What was the outcome of this pregnancy?	◯ Live birth<br>◯ Still birth<br>◯ Miscarriage<br>◯ Termination of pregnancy<br>◯ Other (please specify)
Please tell us how the pregnancy ended?	__________________
How did you deliver?	◯ Vaginal delivery<br>◯ Instrument delivery (i.e. forceps or suction)<br>◯ Elective Caesarean Section<br>◯ Emergency Caesarean Section<br>◯ Other (please specify)
Please tell us about the delivery in this pregnancy	__________________
At how many weeks gestation?	__________________
Could you tell us any more about the pregnancy (i.e. twins, did you use assisted fertility etc.)	______________________
How old were you at your 12th pregnancy (years)?	__________________
What was the outcome of this pregnancy?	◯ Live birth<br>◯ Still birth<br>◯ Miscarriage<br>◯ Termination of pregnancy<br>◯ Other (please specify)
Please tell us how the pregnancy ended?	__________________
How did you deliver?	◯ Vaginal delivery<br>◯ Instrument delivery (i.e. forceps or suction)<br>◯ Elective Caesarean Section<br>◯ Emergency Caesarean Section<br>◯ Other (please specify)
Please tell us about the delivery in this pregnancy	__________________
At how many weeks gestation?	__________________
Could you tell us any more about the pregnancy (i.e. twins, did you use assisted fertility etc.)	______________________

How old were you at your 13th pregnancy (years)?	__________________________________
What was the outcome of this pregnancy?	◯ Live birth<br>◯ Still birth<br>◯ Miscarriage<br>◯ Termination of pregnancy<br>◯ Other (please specify)
Please tell us how the pregnancy ended?	__________________________________
How did you deliver?	◯ Vaginal delivery<br>◯ Instrument delivery (i.e. forceps or suction)<br>◯ Elective Caesarean Section<br>◯ Emergency Caesarean Section<br>◯ Other (please specify)
Please tell us about the delivery in this pregnancy	__________________________________
At how many weeks gestation?	__________________________________
Could you tell us any more about the pregnancy (i.e. twins, did you use assisted fertility etc.)	____
How old were you at your 14th pregnancy (years)?	__________________________________
What was the outcome of this pregnancy?	◯ Live birth<br>◯ Still birth<br>◯ Miscarriage<br>◯ Termination of pregnancy<br>◯ Other (please specify)
Please tell us how the pregnancy ended?	__________________________________
How did you deliver?	◯ Vaginal delivery<br>◯ Instrument delivery (i.e. forceps or suction)<br>◯ Elective Caesarean Section<br>◯ Emergency Caesarean Section<br>◯ Other (please specify)
Please tell us about the delivery in this pregnancy	__________________________________
At how many weeks gestation?	__________________________________
Could you tell us any more about the pregnancy (i.e. twins, did you use assisted fertility etc.)	____

How old were you at your 15th pregnancy (years)?	__________________________
What was the outcome of this pregnancy?	◯ Live birth<br>◯ Still birth<br>◯ Miscarriage<br>◯ Termination of pregnancy<br>◯ Other (please specify)
Please tell us how the pregnancy ended?	__________________________
How did you deliver?	◯ Vaginal delivery<br>◯ Instrument delivery (i.e. forceps or suction)<br>◯ Elective Caesarean Section<br>◯ Emergency Caesarean Section<br>◯ Other (please specify)
Please tell us about the delivery in this pregnancy	__________________________
At how many weeks gestation?	__________________________
Could you tell us any more about the pregnancy (i.e. twins, did you use assisted fertility etc.)	__________________________________
Have you ever tried to fall pregnant?	◯ Yes<br>◯ No
Have you ever been unable to conceive after 1 year of regular (every 2-3 days), unprotected intercourse?	◯ Yes<br>◯ No
How old were you at this time (when you started) ?	__________________________
Have you ever been unable to conceive after more than 2 years of regular (every 2-3 days), unprotected intercourse?	◯ Yes<br>◯ No
Have you ever had any surgery to any gynaecological organs (e.g. vagina, uterus, ovaries and fallopian tubes) ?	◯ Yes<br>◯ No
Please give details:	__________________________________
Have you or your partner ever been evaluated or treated for fertility problems or miscarriage in the past?	◯ Yes<br>◯ No
Please give details:	__________________________________

Have you ever been advised by a doctor to try any fertility-enhancing drugs?	◯ Yes<br>◯ No
Have you ever used any fertility-enhancing drugs?	◯ Yes<br>◯ No
Please give details:	__________________________________
Have you ever been advised by a doctor to try artificial insemination?	◯ Yes<br>◯ No
Have you ever tried artificial insemination?	◯ Yes<br>◯ No
Please give details:	__________________________________
Have you ever been advised to try IVF (in-vitro fertilisation) or ICSI (intra-cytoplasmic sperm injection)?	◯ Yes<br>◯ No
Have you ever attempted IVF (in-vitro fertilisation) or ICSI (intra-cytoplasmic sperm injection)?	◯ Yes<br>◯ No
Please give details:	__________________________________
Have you ever had an ultrasound scan of your pelvis?	◯ Yes<br>◯ No
What was the outcome of your ultrasound?	__________________________________
Have you ever had a laparoscopy (key-hole surgery)?	◯ Yes<br>◯ No
What was the outcome of your laparoscopy?	__________________________________
Have you ever had a salpingogram (dye test of the fallopian tubes) ?	◯ Yes<br>◯ No
What was the outcome of your salpingogram?	__________________________________
Have you ever applied for adoption?	◯ Yes<br>◯ No

Do you have any adopted children?
◯ Yes
◯ No

How many adopted children do you have?
____

Is there anything regarding your sexual, menstrual or fertility history that we have not asked that you would like to tell us about?
____

Thank you for completing the questionnaire so far, the final part of the questionnaire is to complete the FSFI (female sexual function index), which is a validated questionnaire exploring female sexual function.

INSTRUCTIONS: These questions are about your sexual feelings and responses during the past 4 weeks. Please answer the following questions as honestly and clearly as possible. Your responses will be kept completely confidential. In answering these questions, the following definitions apply:

Sexual activity can include caressing, foreplay, masturbation and vaginal intercourse.

Sexual intercourse is defined as penile penetration (entry) of the vagina.

Sexual stimulation includes situations like foreplay with a partner, self-stimulation (masturbation) or sexual fantasy.

Are you happy to answer questions about your sexual functioning?
◯ Yes
◯ No

Sexual desire or interest is a feeling that includes wanting to have a sexual experience, feeling receptive to a partner's sexual initiation and thinking or fantasising about having sex.

Over the past 4 weeks, how often have you felt sexual desire or interest?
◯ Almost Always or Always
◯ Most of the time (more than half the time)
◯ Some of the time (about half the time)
◯ Occasionally (less than half the time)
◯ Almost Never or Never

Over the past 4 weeks, how would you rate your level (degree) of sexual desire or interest?
◯ Very High
◯ High
◯ Moderate
◯ Low
◯ Very Low or None at all

Sexual arousal is a feeling that includes both the physical and mental aspects of sexual excitement. It may include feelings of warmth or tingling in the genitals, lubrication (wetness), or muscle contractions.

Over the past 4 weeks, how often have you felt sexually aroused ("turned on") during sexual activity or intercourse?	◯ No Sexual Activity<br>◯ Almost always or always<br>◯ Most of the time (more than half the time)<br>◯ Some of the time (about half the time)<br>◯ Occasionally (less than half the time)<br>◯ Almost never or never
Over the past 4 weeks, how often have you felt sexually aroused ("turned on") during sexual activity or intercourse?	◯ No Sexual Activity<br>◯ Very High<br>◯ High<br>◯ Moderate<br>◯ Low<br>◯ Very Low or None at all
Over the past 4 weeks, how confident have you felt about becoming sexually aroused during sexual activity or intercourse?	◯ No Sexual Activity<br>◯ Very Highly Confident<br>◯ Highly Confident<br>◯ Moderately Confident<br>◯ Slightly Confident<br>◯ Very slightly or not confident
Over the past 4 weeks, how often have you been satisfied with your arousal (excitement) during sexual activity or intercourse?	◯ No Sexual Activity<br>◯ Almost always or always<br>◯ Most of the time (more than half the time)<br>◯ Some of the time (about half the time)<br>◯ Occasionally (less than half the time)<br>◯ Almost never or never
Over the past 4 weeks, how often have you become lubricated ("wet") during sexual activity or intercourse?	◯ No Sexual Activity<br>◯ Almost always or always<br>◯ Most of the time (more than half the time)<br>◯ Some of the time (about half the time)<br>◯ Occasionally (less than half the time)<br>◯ Almost never or never
Over the past 4 weeks, how difficult has it been to become lubricated ("wet") during sexual activity or intercourse?	◯ No Sexual Activity<br>◯ Not difficult<br>◯ Slightly Difficult<br>◯ Difficult<br>◯ Very Difficult<br>◯ Extremely Difficult or Impossible
Over the past 4 weeks, how often have you maintained your lubrication ("wetness") until the completion of sexual activity or intercourse?	◯ No Sexual Activity<br>◯ Almost always or always<br>◯ Most of the time (more than half the time)<br>◯ Some of the time (about half the time)<br>◯ Occasionally (less than half the time)<br>◯ Almost never or never
Over the past 4 weeks, how difficult has it been to maintain your lubrication ("wetness") until the completion of sexual activity or intercourse?	◯ No Sexual Activity<br>◯ Not difficult<br>◯ Slightly Difficult<br>◯ Difficult<br>◯ Very Difficult<br>◯ Extremely Difficult or Impossible

Over the past 4 weeks, when you had sexual stimulation or intercourse, how often have you reached an orgasm (climax)?	◯ No Sexual Activity<br>◯ Almost always or always<br>◯ Most of the time (more than half the time)<br>◯ Some of the time (about half the time)<br>◯ Occasionally (less than half the time)<br>◯ Almost never or never
Over the past 4 weeks, when you have had sexual stimulation or intercourse, how difficult has it been for you to reach an orgasm (climax)?	◯ No Sexual Activity<br>◯ Not difficult<br>◯ Slightly Difficult<br>◯ Difficult<br>◯ Very Difficult<br>◯ Extremely Difficult or Impossible
Over the past 4 weeks, how satisfied have you been with your ability to reach an orgasm (climax) during sexual activity or intercourse?	◯ No Sexual Activity<br>◯ Very Satisfied<br>◯ Satisfied<br>◯ Neither Satisfied/Dissatisfied<br>◯ Dissatisfied<br>◯ Very Dissatisfied
Over the past 4 weeks, how satisfied have you been with the amount of emotional closeness between you and your partner during sexual activity?	◯ No Sexual Activity<br>◯ Very Satisfied<br>◯ Satisfied<br>◯ Neither Satisfied/Dissatisfied<br>◯ Dissatisfied<br>◯ Very Dissatisfied
Over the past 4 weeks, how satisfied have you been with your sexual relationship with your partner?	◯ No Sexual Activity<br>◯ Very Satisfied<br>◯ Satisfied<br>◯ Neither Satisfied/Dissatisfied<br>◯ Dissatisfied<br>◯ Very Dissatisfied
Over the past 4 weeks, how satisfied have you been with your overall sex life?	◯ No Sexual Activity<br>◯ Very Satisfied<br>◯ Satisfied<br>◯ Neither Satisfied/Dissatisfied<br>◯ Dissatisfied<br>◯ Very Dissatisfied
Over the past 4 weeks, how often have you experienced discomfort or pain during vaginal penetration?	◯ Did not attempt intercourse<br>◯ Almost never or never<br>◯ Occasionally (less than half the time)<br>◯ Some of the time (about half the time)<br>◯ Most of the time (more than half the time)<br>◯ Almost always or always
Over the past 4 weeks, how often have you experienced discomfort or pain after vaginal penetration?	◯ Did not attempt intercourse<br>◯ Almost never or never<br>◯ Occasionally (less than half the time)<br>◯ Some of the time (about half the time)<br>◯ Most of the time (more than half the time)<br>◯ Almost always or always

Over the past 4 weeks, how would you rate your level (degree) of discomfort or pain during or after vaginal penetration?	◯ Did not attempt intercourse<br>◯ Very Low or None at all<br>◯ Low<br>◯ Moderate<br>◯ High<br>◯ Very High
Is the anything further about your recent sexual activity you would like to share?	______________________________

This conclude the questionnaire, thank you for taking part in our study.
